# Draft Genome Sequence of *Halomonas* sp. Strain SL1, a Putative Polyhydroxyalkanoate-Producing Halophile

**DOI:** 10.1128/MRA.00915-18

**Published:** 2019-01-03

**Authors:** Tyler Rommel, Kazuhiko Kurosawa, Lucy M. McCully, Mark W. Silby, Vanni Bucci, Christopher J. Brigham

**Affiliations:** a Department of Bioengineering, University of Massachusetts Dartmouth, Dartmouth, Massachusetts, USA; b Department of Biology, Massachusetts Institute of Technology, Cambridge, Massachusetts, USA; c Department of Biology, University of Massachusetts Dartmouth, Dartmouth, Massachusetts, USA; Louisiana State University

## Abstract

Halomonas sp. strain SL1, a halophilic gammaproteobacterium, was isolated from samples from the Great Salt Lake in Utah. We report here the draft genome sequence of SL1, which has an estimated total sequence length of 3.6 Mb.

## ANNOUNCEMENT

Halomonas species are a diverse group that includes plant and human pathogens and other soil-dwelling organisms, such as Halomonas halophila, Halomonas elongata, and Halomonas salina (1). Halomonas spp. and related species synthesize and mobilize poly(3-hydroxybutyrate) [P(3HB)] and other polyhydroxyalkanoate (PHA) biopolymers as part of their natural metabolism (2). PHA has attracted much recent interest as a potential bio-based biodegradable alternative to petroleum-based plastics (3). Halophiles are of particular interest in PHA production due to lower fermentation costs (4).

Halomonas sp. strain SL1 was isolated from sediment from the Great Salt Lake in Utah using LB agar (Lennox) plates supplemented with 10% (wt/vol) NaCl, adjusted to pH 8.5. This medium was used for all experiments in this study. The bacterium grows using high-salt medium (5 to 20% [wt/vol]). Genomic DNA of Halomonas sp. SL1 was extracted using the Wizard genomic DNA puriﬁcation kit (Promega, Madison, WI, USA). Libraries were then generated using the Nextera XT sequencing kit (Illumina, San Diego, CA, USA) and sequenced by an Illumina MiSeq platform at the Tufts University Genomics Core Facility. We obtained 2,535,240 2 × 250-bp reads (185-fold coverage), which were assembled into 41 contigs (from 527 to 478,058 bp) using CLC Genomics Workbench version 10.1.1. The contig *N*_50_ value is 175,347 bp. The assembler was configured with a minimum contig length of 500 bp, a bubble size of 98, and a word size of 45.

The draft genome sequence of SL1 comprises 3,649,259 bp (67.6% G+C content) and was annotated using the NCBI Prokaryotic Genome Annotation Pipeline (PGAP). We predicted 3,375 protein-coding sequences, 58 tRNA sequences, and at least one copy each of 16S and 23S rRNA genes. The 16S rRNA gene sequence of SL1 was found to be most closely related to that of Halomonas halophila strain CCM 3662 (99% identity based on nucleotide BLAST using the RDP Classifier [5]).

In a BLAST search of the assembled contigs, we detected two putative β-ketothiolase (*phaA*) genes (contigs 1 [locus_tag=C9J49_00100] and 5 [locus_tag=C9J49_001965]), two putative acetoacetyl-coenzyme A (acetoacetyl-CoA) reductase (*phaB*) genes (contigs 19 [locus_tag=C9J49_013185] and 28 [locus_tag=C9J49_016095]), and a putative PHA polymerase (*phaC*) gene (contig 13 [locus_tag=C9J49_008420]). PGAP annotation identified a putative regulatory gene (*phaR*) in contig 10 (locus_tag=C9J49_003675). These ﬁndings suggest that SL1 can synthesize intracellular polyhydroxyalkanoates. However, genes for the PHA-degrading enzyme *phaZ* were not detected.

### Data availability.

This whole-genome shotgun sequencing project has been deposited in DDBJ/ENA/GenBank under accession number PYUQ00000000. The version described in this paper is the second version, PYUQ02000000. Raw sequence reads have been deposited in the NCBI Sequence Read Archive under accession number PRJNA445724.
